# Lactic Acid Regulation: A Potential Therapeutic Option in Rheumatoid Arthritis

**DOI:** 10.1155/2022/2280973

**Published:** 2022-08-24

**Authors:** Qianlei Wang, James Asenso, Ning Xiao, Jinzhang Gao, Feng Xiao, Jiajie Kuai, Wei Wei, Chun Wang

**Affiliations:** ^1^Institute of Clinical Pharmacology, Anhui Medical University, Hefei, China; ^2^Key Laboratory of Anti-Inflammatory and Immune Medicine, Ministry of Education, Hefei, China; ^3^Anhui Collaborative Innovation Centre of Anti-Inflammatory and Immune Medicine, Hefei, China; ^4^Center of Rheumatoid Arthritis of Anhui Medical University, Hefei, China; ^5^Department of Pharmaceutical Sciences, Sunyani Technical University, P.O. Box 206 Sunyani, Ghana

## Abstract

Rheumatoid arthritis (RA) is a chronic, persistent autoimmune disease that causes severe joint tissue damage and irreversible disability. Cumulative evidence suggests that patients suffering from RA for long durations are at risk of functional damage to cardiovascular, kidney, lung, and other tissues. This seriously affects the quality of work and life of patients. To date, no clear etiology of RA has been found. Recent studies have revealed that the massive proliferation of synoviocytes and immune cells requires a large amount of energy supply. Rapid energy supply depends on the anaerobic glucose metabolic pathway in both RA animal models and clinical patients. Anaerobic glycolysis can increase intracellular lactic acid (LA) content. LA induces the overexpression of monocarboxylate transporters (MCTs) in cell membranes. MCTs rapidly transport LA from the intracellular to the intercellular or articular cavity. Hence, a relatively high accumulation of LA could be formed in the intercellular and articular cavities of inflammatory joints. Moreover, LA contributes to the migration and activation of immune cells. Immune cells proliferate and secrete interleukins (IL) including IL-1, IL-2, IL-13, IL-17, and other inflammatory factors. These inflammatory factors enhance the immune inflammatory response of the body and aggravate the condition of RA patients. In this paper, the effects of LA on RA pathogenesis will be summarized from the perspective of the production, transport, and metabolism of synoviocytes and immune cells. Additionally, the drugs involved in the production, transport, and metabolism of LA are highlighted.

## 1. Introduction

Rheumatoid arthritis (RA) is a common chronic disease that can lead to joint damage and irreversible severe disability. RA and its treatment with drugs increase the risk of kidney [1] and cardiovascular diseases [2, 3], which often increase chronic pain and add to patients' social burdens. Thus, RA greatly affects the quality of life of patients. However, the exact cause of RA has not been fully elucidated. Symptoms of RA, such as facet joint polyarthritis of the hands and feet, morning stiffness, and other physical symptoms [4], can lead to synovial hyperplasia and progressive joint destruction in patients. Early treatment is highly recommended for the effective management of RA. Consequently, research efforts on rheumatoid joints to uncover potential biomarkers, including lactic acid (LA) [5], T cell quantity, plasma and slide microRNAs [6], and C-reactive proteins [7], have received significant attention in recent years.

Synovial hyperplasia in RA patients requires considerable energy. Hypoxia is a basic metabolic change in many inflammatory diseases, and adenosine triphosphate (ATP) supply needs to be provided by the anaerobic glycolysis pathway to maintain the energy requirements for high-level cell proliferation. This assertion is consistent with RA studies that have observed elevated lactate levels and decreased glucose in the synovial fluid [8–10]. It has been reported that enhanced glycolytic activity and increased LA and pyruvate promote angiogenesis and pannus formation [5], leading to the destruction of joint tissue structure in RA. Additionally, the pH value of synovial fluid decreases with increasing LA under hypoxic conditions, suggesting a positive correlation between LA and pH and pO_2_ [11]. These observations have been corroborated by increased lactate levels in synovial fluid extracted from the joint cavity of RA patients [12–14]. The effect of LA in rapidly growing tissues is even more evident in tumors. Not only does the LA content significantly increase in tumor tissues but the secretion of vascular endothelial growth factor (VEGF) also increases [15–17]. VEGF promotes angiogenesis [15] and supplies energy for tumor growth. In addition, LA induces the secretion of hyaluronic acid to promote tumor development [18]. Thus, studies on the involvement of LA in the pathogenesis of RA and other inflammatory conditions are warranted.

An increase in LA has been associated with an increase in the expression of monocarboxylate transporter (MCT) family members, which consequently affects the transport and metabolism of LA. In RA, chondrocytes are sensitive to changes in extracellular acidity, and the increase in LA affects stromal regeneration [19]. Tissue acidosis is also an important feature of RA [20]. LA contributes to the activation and migration and simultaneously stimulates the proliferation of proinflammatory cells such as tumor necrosis factor (TNF), and interleukin (IL) secreted by immune cells. TNF and IL-1 are the main inflammatory factors in RA. LA stimulates the transcriptional activation of TNF and IL-1 by stimulating the transcriptional activity of nuclear factor-*κ*B (NF-*κ*B). Although LA is less effective than lipopolysaccharide (LPS), the two exert a synergistic effect. In addition, LA serves an important role with respect to myeloid differentiation factor-2 (MD-2), stimulation of the Toll-like receptor 4 (TLR4) community, and activation of inflammatory genes in human U937 tissue cells [21]. Sodium lactate and LPS have also been reported to exert synergistic effects on the activities of proinflammatory transcription factors activating protein-1 and NF-*κ*B [22]. These findings suggest that LA plays a pivotal role in the activity of immune cells and the secretion of cytokines in the synovium of RA.

## 2. Production, Transport, and Metabolism of Lactic Acid

**Figure 1:**
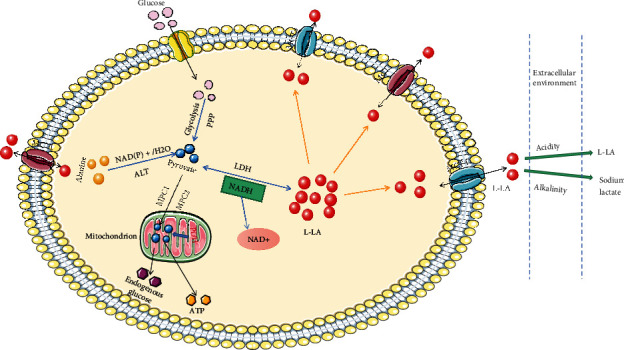
Pyruvate derived from glucose and alanine generates LA under the action of LDH. LA is transported by MCT to maintain the homeostasis of the intracellular microenvironment. The presence of LA outside the cell depends on the pH of the extracellular environment. LA is converted into pyruvate by the reverse action of LDH. MCP1 and MCP2 in the cytoplasm transport pyruvate to mitochondria and are metabolized by PDH into endogenous glucose and ATP. PPP: pentose phosphate pathway; NAD(P)^+^: nicotinamide adenine dinucleotide (phosphate); ALT: alanine aminotransferase; MPC: mitochondrial pyruvate transporter; PDH: pyruvate dehydrogenase; LDH: lactate dehydrogenase.

LA exists in the body largely as two enantiomers L-LA and D-LA. While L-LA is a common product of the body's metabolism, D-LA is scarcely produced by some microorganisms [23]. In humans, L-LA mainly originates from the conversion of glucose and alanine into pyruvate [24]. Glucose forms pyruvate through glycolysis and the pentose phosphate pathway. Under the action of lactate dehydrogenase (LDH), pyruvate is reduced to L-LA, while NADH is oxidized to NAD+ [25]. This is a reversible reaction. LA is not only a source of fuel for cells but may also play a role in some diseases and the regulation of cell function [26–28]. It is important to mention that the form of LA depends on the pH of the inflammatory microenvironment [29] (Figure 1).

MCTs are important transporters that are crucial for the intracellular production and transmembrane transport of LA. The transfer of LA into or out of cells by MCT1 depends on the metabolic status of cells [30]. Similarly, the transport of LA by MCT4 is bidirectional and mostly tends to transport LA to the outside of the cell during glycolysis thereby reducing the intracellular concentration of LA [31, 32] (Figure 1). Notwithstanding, this seems not to significantly increase the extracellular LA concentration, which may be related to bicarbonate in synovial fluid. Other subtypes of MCT, such as MCT2, are mainly expressed in neurons and promote the utilization of LA and ketone bodies as energy substrates [33]. Furthermore, MCT3 is thought to be largely expressed in the epithelia of the retinal pigment and choroid plexus, promoting the transport of LA [34].

During the process of LA metabolism, L-LA is oxidized to pyruvate by LDH, which is the first step of metabolism. The role of LDH in the conversion between pyruvate and LA is bidirectional; hence, the regulation of LDH activity needs to be controlled within a certain range. Pyruvate is transported to the mitochondria by the cytoplasmic proteins MPC1 and MPC2 [35] (Figure 1). The overexpression of MPC1 and MPC2 can significantly reduce pyruvate and the proliferation of cancer cells [36, 37], which provides guiding significance to the study of inhibiting the proliferation of RA synoviocytes. Pyruvate is metabolized to ATP and endogenous glucose in mitochondria by the pyruvate dehydrogenase (PDH) complex and pyruvate carboxylase by the oxidation and dextran pathways, respectively.

## 3. Lactic Acid and Synoviocytes

The proliferation of synoviocytes is one of the most important pathological features of RA. The rapid proliferation of RA synovial fibroblasts (RASFs) could result in higher intracellular LA concentrations and induce the activation and overexpression of MCTs on the cell membrane. Efflux of LA leads to acidification of synovial fluid in RA patients [13]. In a study of bovine fibroblast-like synoviocytes, D-LA was found to promote the expression and secretion of the proinflammatory factors IL-6 and IL-8 through intracellular transport by MCT1 via the induction of the ERK1/2, P38, Akt, and NF-*κ*B signaling pathways, leading to an inflammatory response [38]. In another study, it was observed that D-LA could disrupt the metabolism of bovine fibroblast synoviocytes and promote glycolysis and gluconeogenesis and pyruvate and galactose metabolism [39]. These findings indicate that LA can act on the signaling pathway of synoviocytes and that a high extracellular concentration of LA could promote further production of LA in synoviocytes.

## 4. Lactic Acid and Immune Cells

### 4.1. T Cells

LA regulates the differentiation of T cells and the secretion of inflammatory cytokines. On the extracellular side, the chemokine receptor CXCR3 binds to the chemokine CXCL10 and interferes with glycolysis. The resulting sodium lactate inhibits the movement of CD4+ T cells and induces the conversion of CD4+ T helper cells to the Th17 subset while promoting CD4+ T-cell-induced secretion of the proinflammatory cytokine IL-17 [12]. The increase in Th17 cells causes an immune imbalance of Th17/Treg cells. A study has shown that IL-2 levels in the serum of RA patients correlate not only with disease activity and autoantibody levels but also with Th17/Treg immune imbalance [40]. Additionally, LA has been demonstrated to increase IL-2-specific mRNA expression and IL-2 activity in mitogen-stimulated T cell cultures [41]. In a study using HUT-78T cells, LA promoted the production of IL-4 and IL-13 through MCTs to affect the activation process of T cells; thus, the effect of LA on the expression and secretion of IL-13 was more significant than that of IL-4 [42]. As an important carbon source in the Krebs cycle, LA provides energy for cell migration and movement. This suggests that any process that inhibits the uptake of LA in CD4+ T cells consequently reduces the supply of carbon to the intracellular mitochondrial Krebs cycle. For instance, the metastatic movement of CD4+ T cells is reduced, and these cells accumulate at the site of inflammation, while excessive secretion of inflammatory factors by CD4+ T cells promotes the inflammatory process [43].

Most CD8+ T cells in RA synovium are located around ectopic lymph follicles or vessels. In the synovial microenvironment of hypoxia and malnutrition, the proliferation function of activated CD8+ T cells is dominated by glycolysis. Activated CD8+ T cells release proinflammatory and cytolytic mediators to promote RA [44]. The supply of H+ is necessary for LA to regulate the motility of CD8+ T cells. Interestingly, LA inhibition of CD8+ T cell motility is independent of glycolysis control [12].

**Figure 2:**
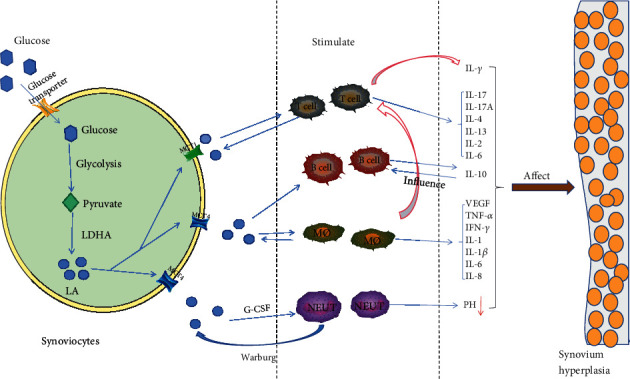
Synoviocytes, T cells, macrophages, and neutrophils in RA in a hypoxic microenvironment. Glycolysis produces a large amount of LA through transporters and accumulates in inflammatory tissues to reduce synovial fluid pH. LA stimulates immune cells to activate, proliferate, and secrete inflammatory stimulating factors, which promotes synovial cell proliferation and leads to joint tissue damage and even disability. LDHA: lactate dehydrogenase; MCT1: monocarboxylic acid transporter 1; MCT4: monocarboxylic acid transporter 4; G-CSF: granulocyte colony stimulating factor; MØ: macrophages; NEUT: neutrophils; IL: interleukin; TNF-*α*: tumor necrosis factor-alpha.

Studies in autoimmune diseases have shown that T cells in low-oxygen environments do not produce as much ATP and LA as healthy control T cells; however, they can proliferate [45]. Even when oxygen is available, T cells convert pyruvate to LA rather than acetyl-CoA due to a special glucose metabolism process known as aerobic glycolysis or the Warburg effect [46]. In RA, a large number of activated T helper cells activate Saccharomyces activity in synovial fibroblasts (SF) and induce the transition of SF to aerobic glycolysis. High secretion of LA and IL-6 in SF triggers the inflammatory phenotype of SF [47]. Reports indicate that RASFs can stimulate Th17 cells to increase the expression of IL-17A, hence making RASFs one of the targets of IL-17A. Additionally, RASFs interact with Th17 cells to induce IL-6 production; thus, RASFs and Th17 cells activate each other in a proinflammatory feedback loop, leading to increased expression of IL-6 and IL-17A [48–51] (Figure 2). This persistent harmful feedback loop between T cells and RASFs promotes inflammation and persistent joint damage.

### 4.2. Macrophages

Macrophages have two polarized forms: M1 and M2. M1-polarized macrophages are proinflammatory and joint destructive and secrete important inflammatory factors such as TNF-*α* and IL-1. On the other hand, M2-polarized macrophages exert anti-inflammatory and immunomodulatory effects and serve key roles in tissue repair, remodeling, and fibrosis [52, 53]. In addition, it has been found that umbilical cord-derived human mesenchymal stromal cells (UC-MSCs) accumulate at inflammatory sites and that UC-MSCs can induce an increase in the local concentration of LA, which could potentially promote the production of M2 macrophages [54].

Large numbers of macrophages in the presence of ganglionic inflammatory sites in RA have been reported [55]. Existing studies have found that effective treatments for arthritis can reduce the number of macrophages in arthritic joints [56]. Additionally, the metabolic state of macrophages shows an increase in glycolysis in response to LA [57]. Moreover, LA enhances LPS-stimulated expression of inflammatory genes such as IL-1*β*, IL-6, and IL-8 in macrophages and promotes LPS-induced activation of TLR4 in NF-*κ*B signaling [21]. Furthermore, LA can increase the expression of VEGF mRNA and VEGF proteins in THP-1 macrophages [58], leading to the formation of blood vessels at inflammatory sites and providing a nutritional guarantee for tissue proliferation. Together, these findings show that the effects of LA on macrophage metabolism and gene expression may promote the evolution and development of RA.

Studies on lactate transporters in macrophages have found that MCT4 is upregulated by TLR2 and TLR4 agonists in all types of macrophages except TLR3. The upregulation of MCT4 and the transport of LA to the outside of cells are necessary mechanisms for macrophages to maintain hyperglycolysis [59], and this mechanism is crucial to fully activate an inflammatory response. In RA, macrophages are different from T cells. T cells proliferate exponentially due to a lack of energy, while macrophages maintain an energy supply by taking up large amounts of glucose. In another study, the ability of LA to enhance the differentiation of Th1 cells and the secretion of interferon-*γ* through macrophages associated with tumor tissue was highlighted [60] (Figure 2). In the pathological state of RA, both macrophages and T cells exist in the inflammatory sites of joints, and the interaction between macrophages and T cells by LA may have a specific effect on inflammation.

### 4.3. B Cells

Overactivated B cells are thought to be harmful in RA. This is because the depletion of mature B cells significantly reduces disease severity in the CIA model [61]. However, studies in RA mice have shown that regulation of B cells has potential protective and therapeutic potential [62, 63]. The anti-inflammatory cytokine IL-10 determines the regulatory effect of B cells [64]. For example, activated spleen B cells led to an in vitro increase in the secretion of IL-10 in apoptotic cells while inhibiting IL-10 in vivo, thus reversing the beneficial effect of apoptotic cell therapy [62]. Therefore, the cytokine IL-10 secreted by B cells serves an immunomodulatory role in autoimmune diseases [65] (Figure 2).

Studies on the expression of LDHA in B lymphoma showed that LDHA5 expression is highly upregulated to promote the conversion of pyruvate to LA [66]. As an autocrine factor, LA plays an important role in promoting the growth and proliferation of the Epstein–Barr (EBV) immortalized B lymphoblastic cell line (LCL) under serum-free conditions [67, 68]. Notably, LA was observed to stimulate B cells to the maximum extent at a concentration of 2.4-4.8 mM [68]. In addition, EBV-immortalized LCL can produce a large amount of LA in the extracellular environment [69].

Anticitrullinated protein antibodies (ACPAs) are a hallmark of preattack RA examination and have very high diagnostic specificity—they are present throughout the disease course [70, 71]. Studies have shown that ACPA plasma blasts may migrate to the synovial compartment. When the microenvironment permits, these cells can survive long term and secrete ACPAs [72]. Moreover, citrulline-responsive B cells can migrate to the synovium for differentiation early in development [73]. After the onset of RA, ACPA secretion is further increased and promotes various inflammatory processes in RA [74]. ACPA FC-glycans play a role in inflammatory modulation and are involved in the transition from asymptomatic autoimmunity to inflammatory arthritis through IL-23 and T-helper 17 cells [75], but the exact mechanism by which Th17 cells are involved in this process remains unclear. The clinical therapeutic effect for patients with ACPA-positive RA is greater than that for patients with ACPA-negative RA [76]. These results suggest that ACPA-producing B cells indeed play an important role in the inflammatory process of RA. From the above, we know that sodium lactate can induce the conversion of CD4+ T helper cells to the Th17 subset, and we speculated that LA may indirectly participate in the proinflammatory process of ACPAs. Among RA studies, there is a lack of existing research on the interactions between lactic acid and ACPAs or ACPA-producing B cells, so this is a worthy direction of future research.

### 4.4. Neutrophils

Neutrophil mobilization is a key feature of the acute inflammatory response. Studies have shown that D-LA promotes the adhesion of bovine neutrophils to endothelial cells and the formation of neutrophil extracellular traps (NETs) through plasma membrane transport of MCT1 and triggers NETosis in a PAD4- and MCT1-dependent manner. In addition, LA has been found to enhance neutrophil mobilization by increasing vascular permeability and chemokines (such as G-CSF) [77]. Mobilized neutrophils are involved in the pathogenesis of autoimmune diseases, including RA, mainly through the formation of NETs [78]. NETs are mainly composed of histones and DNA fibers [79], and in the formation of NETs, the extracellular extrusion of DNA, histones, and other proteins stimulates the immune response, which leads to inflammation and joint injury [80]. In a recent study, the formation and metabolism of NETs were investigated using two types of NOX-dependent NET inducer, PMA, and the non-NOX-dependent NET inducer A23187. Both inducers were found to increase the extracellular acidification rate of neutrophils. These results revealed that the activities of LDH, pyruvate kinase M2 (PKM2) dimerization, and PKM2 decreased, similar to the effects of T cells, which promoted the production of LA through the Warburg effect. The study also found that human neutrophils were treated with exogenous LA to form NETs, by inhibiting the activity of LDH [81]. These findings further highlight the assertion that LA contributes to the formation of neutrophilic NETs and that LA is involved in neutrophil-mediated autoimmune diseases.

### 4.5. Other Immune Cells

In addition to the immune cells mentioned above, studies on the stimulation of rat dendritic cells (DCs) with TLR agonists revealed increased glycolysis and LA, which were associated with the acquisition of immune stimulatory function [82]. In another study, GM-CSF-derived DCs expressed iNOS and produced NO in mice after TLR agonist stimulation, which inhibited OXPHOS and further promoted glycolysis [83]. Furthermore, research on tumor tissues has revealed that high concentrations of LA can induce tumor-specific DCs either alone or in combination with cytokines [84]. LA inhibits LPS-induced mast cell activation and LPS-induced cytokine production by inhibiting glycolysis in vivo [85]. Importantly, cumulative evidence has demonstrated a significant relationship and role between LA and many immune cells in inflammatory and immune diseases which warrants further studies to explore the corresponding therapeutic potential.

## 5. Related Drugs Affecting Lactic Acid

### 5.1. Lactic Acid Transporter-Related Drugs

Excessive LA is transported through the overexpression of MCT to maintain the normal metabolic environment of cells and provide constant energy demand for cell proliferation and cytokine secretion. Inhibition of MCTs controls the transport of LA and affects the abnormal metabolism of immune cells and synoviocytes. Thus, MCT could be a therapeutic target for drug development for the treatment of RA.

AR-C155858, AstraZeneca's MCT1, and MCT2 dual specific inhibitors reportedly inhibit lactate outflow and thereby inhibit lymphocyte activation which contributes to the improvement of RA disease [86–88]. Additionally, AR-C155858 effectively inhibits the proliferation of T cells, which supports the hypothesis that the activation and proliferation of T cells is a highly glycolytic process maintained by the rapid permeation of LA [86]. Another inhibitor, AS2495674, preferentially inhibits MCT1 in activated T cells, resulting in intracellular lactate accumulation, reduced glycolytic flux, and limited lymphocyte proliferation [89]. This has a positive effect on inflammatory immune diseases.

A highly selective and noncompetitive MCT4 inhibitor, bindarit, was found in recent studies, and its selectivity for MCT4 was 15 times higher than that for MCT1, which exhibited strong inhibition against the uptake of L-LA through MCT4 [90].

Most of the drugs that inhibit MCTs are derived from drugs originally used to treat tumors. For example, the antitumor drug 7-alkylamino 3-carboxycoumarin (7ACC) can control the entry and exit of LA in cells expressing MCT1 or MCT4. Interestingly, in cells expressing MCT1 and MCT4 simultaneously, 7ACC restricts the entry of LA into cells but does not affect the outflow of LA [91]. Lonidamine, as an antitumor drug, has been found to inhibit the mitochondrial pyruvate transporter (MPC), mitochondrial productivity pathway, and extracellular transport of LA by suppressing MCT [92, 93].

Alpha-cyano-4-hydroxycinnamic acid (*α*-CHCA), a classic MCT inhibitor, can block LA-enhanced inflammatory gene expression and inhibit nuclear NF-*κ*B activity in human macrophages [21]. This further reduces the production of downstream inflammatory factors, suppresses inflammation, and ameliorates disease progression.

In addition, traditional NSAIDs such as ibuprofen and salicylic acid can inhibit the uptake of SMCT1 [94]. Other inhibitors of MCTs include phloretin, p-chloromercuribenzene sulfonate, quercetin, and AZD3965 [95, 96]. Thus, MCT inhibitors manifest positive roles in the treatment and improvement of RA through the control of LA transport and accumulation (Table 1).

### 5.2. Drugs Related to Lactic Acid Production and Metabolism

LDHA is a key enzyme in the synthesis of LA. As the functional pathway of glucose metabolism in cells at inflammatory sites is changed, LDHA overexpression leads to a relative increase in the concentrations of LA and lactate. MTX, a typical DMARD, has been an effective and first-line drug in the clinic since it was approved for the treatment of RA in the 1980s [97]. However, in the study by Niitsu et al. [98], it was found that increased levels of LDH were observed in approximately 97% of RA patients treated with MTX.

Through the analysis of immune cells, it was found that the increase in LA is disadvantageous to the treatment of RA. To effectively mobilize hematopoietic stem cells during the treatment of patients with severe RA, the use of filgrastim becomes useful, since it can increase the concentration of LDH twofold [99]. CD8+ T cells (CD8) can release proinflammatory and cellular decomposition mediators in normal inflammation [100]. This allows the activation of B cell differentiation and proinflammatory expression [101] and promotes the development of RA. The activity of CD8 in RA is dependent on LDHA activity, and the activation of B cells is also affected by LDHA activity. For instance, the inhibitor FX11 has been reported to suppress the inflammatory ability of CD8+ and B cells via the inhibition of LDHA [44]. Additionally, it has been found that another LDHA inhibitor, GSK2837808A, can maintain normal cell viability without affecting other common enzymes and ion channels, effectively reducing LDHA levels. It was observed in that same study that the inhibitor can reduce the production of LA and lactate, leading to a reduction in the cytokine IL-1*β* levels [102], resulting in a positive outcome in the treatment of inflammation (Table 2).

Studies on LA metabolism showed that PDH activity can be increased by exercise, phenylbutyric acid, dichloroacetic acid, etc. [103–105]. PDH promotes the conversion of pyruvate to ATP and endogenous glucose. In addition, the PPAR*γ* agonist acetyl coenzyme A and its fragments can promote the activation of pyruvate carboxylase [106, 107]. The processes mentioned above can promote the metabolism of pyruvate and reduce the accumulation of LA by promoting its metabolism.

RA is a condition that requires long-term treatment. Currently, the most commonly used clinical drugs are DMARDs (with MTX as the first choice) followed by steroids, NSAIDs, or biologics [108]. Long-term use of these classes of drugs is associated with adverse reactions that cannot be ignored. Even though later biologic therapies have ushered in the so-called therapeutic revolution, the risk of infection, including bacterial, fungal, and viral infections with biologic agents, cannot be overlooked [109]. Therefore, future experiments targeting alternative treatments with fewer adverse reactions in the treatment of RA are warranted.

## 6. Conclusion

In addition to serving as a tumor marker, growing research evidence has revealed the key roles LA plays in the development of RA and other immune-related conditions. For instance, a low-oxygen environment stimulates glycolysis to produce a large amount of LA, which leads to increased accumulation and retention of immune cells and enhanced secretion of inflammatory factors, resulting in the promotion of inflammation. However, the inhibition of transporter activity can regulate the concentration of LA both inside and outside immune cells. Additionally, inhibition of LDH activity reduces LA generation and promotes the pyruvate metabolic pathway to reduce LA synthesis and accelerate its metabolism. As an important carbon donor in the Krebs cycle, LA is indispensable for the supply of cellular ATP. The clinical significance of LA, particularly, regarding inflammation, makes it a therapeutic target worthy of research attention. Thus, selective and more targeted agents or combinations with other drugs are very important for patients with RA. Importantly, the study of LA in the pathogenesis of RA and other diseases could provide more insights for a new lead for adjuvant therapy for RA.

## Figures and Tables

**Table 1:** Drugs that act on monocarboxylate transporter.

Drug	MCT types	Subjects	Dose	Results
Bindarit	MCT4	Oocytes	Ki (30.2 ± 1.4 *μ*M)	Inhibit the intake of LA [90]
*α*-CHCA	MCTs	U937 cells	2 mM for 30 min	Inhibition of NF-*κ*B activity [21]
Lonidamine (AF-1890)	MCT1, MCT2, MCT4	DB-1 cells DB-1 xenografts in mice	36-40 *μ*M 100 mg/kg, i.p.	Inhibition of pyruvate entry into mitochondria and outflow of L-LA from cells [92, 93]
Ar-C155858	MCT1, MCT2	Rat erythrocytes *Xenopus laevis* oocytes	Ki (2.3 nM) Ki (2 nM)	Inhibition of LA outflow and T cell activation and proliferation [86–88]
AS2495674	MCT1	CD4+ T cells B lymphocytes	EC_50_ (1.2 nM) IC_50_ (0.34 and 0.4 nM)	Intracellular lactate accumulation, glycolysis flux decreased, and lymphocyte proliferation was limited [89]
7ACC	MCT1 MCT4	Cervix cancer cells Female nude mice	IC_50_ (11 nM) 3 mg/kg, i.p.	Control the transfer and efflux of LA [91]
Phloretin	MCT1	*Laevis* oocytes	IC_50_ (17.3 ± 2.37 *μ*M)	Inhibits LA uptake [95]
AZD3965	MCT1	Glycolytic breast cancer cells	250 nM	Inhibit the output of pyruvate and inhibit cell proliferation (cancer cells) [96]
...	...	...	...	...

Note: MCT: monocarboxylate transporter; Ki: inhibitor constant; EC_50_: half effective concentration; DB cells: lymphoma cells; IC_50_: half maximal inhibitory concentration; NF-*κ*B: nuclear factor-*κ*B; i.p.: intraperitoneal injection; LA: lactic acid; 7ACC: 7-alkylamino 3-carboxycoumarins; *α*-CHCA: alpha-cyano-4-hydroxycinnamic acid.

**Table 2:** Drugs that act on the production of lactic acid.

Drug	Target	Subjects	Dose	Results
Methotrexate	Dihydrofolate reductase	RA patients	Less than 20 mg/week or 10 mg/week	The LDH level increased in 97% of patients [98]
Filgrastim	Hematopoietic stem cells	RA patients	10 mg/kg/day	The LDH level increased [99]
FX11	LDHA	CD8+ T cells B cells	8 *μ*M/9 *μ*M	Decreased adipogenesis, migration, proliferation, and effector functions of RA CD8 cells and reduced the transformation of healthy B cells to proinflammatory phenotypes [44]
GSK2837808A	LDHA	Synovial fibroblasts Selected hepatocellular carcinoma cell lines	Less than 20 *μ*M	Decreased production of LDHA and lactate and decreased the cytokine IL-1*β* [102]
...	...	...	...	...

Note: RA: rheumatoid arthritis; LDH: lactate dehydrogenase; IL: interleukin.

## Data Availability

This article is a review article. The graphs and tables included in this article are original and used for the first time in this review.

## Abbreviations

LA: Lactic acid
ATP: Adenosine triphosphate
VEGF: Vascular endothelial growth factor
MCT: Monocarboxylate transporter
TNF: Tumor necrosis factor
IL: Interleukin
NF-*κ*B: Nuclear factor-*κ*B
LPS: Lipopolysaccharide
MD-2: Myeloid differentiation factor-2
TLR4: Toll-like receptor 4
LDH: Lactate dehydrogenase
PDH: Pyruvate dehydrogenase
RASFs: Rheumatoid arthritis synovial fibroblasts
UC-MSCs: Umbilical cord-derived human mesenchymal stromal cells
EBV: Epstein-Barr virus
LCL: Lymphoblastic cell line
NET: Neutrophils extracellular trap
PKM2: Pyruvate kinase M2
DCs: Dendritic cells
MPC: Mitochondrial pyruvate transporter
ACPAs: Anticitrullinated protein antibodies.
